# The Identification of the Banana Endogenous Reference Gene *MaSPS1* and the Construction of Qualitative and Quantitative PCR Detection Methods

**DOI:** 10.3390/genes14122116

**Published:** 2023-11-23

**Authors:** Lili Zhu, Ying Lin, Wenli Yang, Zhiwen Pan, Weiting Chen, Juan Yao, Ou Sheng, Lingyan Zhou, Dagang Jiang

**Affiliations:** 1 College of Life Sciences, South China Agricultural University, Guangzhou 510642, China; 15219204852@163.com (L.Z.); 15875410861@163.com (Y.L.); yang_wenli1998@163.com (W.Y.); panzhiwen@scau.edu.cn (Z.P.); chenweit@126.com (W.C.); yaojuan@scau.edu.cn (J.Y.); 2 College of Agriculture & Biology, Zhongkai University of Agriculture and Engineering, Guangzhou 510225, China; 3 Institute of Fruit Tree Research, Guangdong Academy of Agricultural Sciences, Guangzhou 510640, China; shengou6@126.com

**Keywords:** banana (*Musa paradisiaca*), endogenous reference gene, *MaSPS1*, qPCR

## Abstract

Endogenous reference genes play a crucial role in the qualitative and quantitative PCR detection of genetically modified crops. Currently, there are no systematic studies on the banana endogenous reference gene. In this study, the *MaSPS1* gene was identified as a candidate gene through bioinformatics analysis. The conservation of this gene in different genotypes of banana was tested using PCR, and its specificity in various crops and fruits was also examined. Southern blot analysis showed that there is only one copy of *MaSPS1* in banana. The limit of detection (LOD) test showed that the LOD of the conventional PCR method is approximately 20 copies. The real-time fluorescence quantitative PCR (qPCR) method also exhibited high specificity, with a LOD of approximately 10 copies. The standard curve of the qPCR method met the quantitative requirements, with a limit of quantification (LOQ) of 1.14 × 10^−2^ ng—about 20 copies. Also, the qPCR method demonstrated good repeatability and stability. Hence, the above results indicate that the detection method established in this study has strong specificity, a low detection limit, and good stability. It provides a reliable qualitative and quantitative detection system for banana.

## 1. Introduction

Banana (*Musa paradisiaca*) is one of the world’s most important fruits and the fourth-largest food crop globally [1]. It is highly nutritious, has a high yield, and is widely popular [2]. During the growth and development of bananas, they are subjected to various biotic and abiotic stresses that can reduce their yield and quality [3]. Among the biotic stresses, Fusarium wilt is currently the most harmful disease to production. This disease is caused by the specialized strain of *Fusarium oxysporum* f. sp. *Cubense* [4]. Currently, there are no effective methods for its prevention and control during planting. To solve the challenge posed by Fusarium wilt, genetic engineering techniques, including gene-editing technologies, are considered viable options [5]. There have been multiple reports that genetically modified (GM) bananas show enhanced resistance to biotic and abiotic stresses [6,7].

In April 2023, the Philippines officially exempted gene-edited bananas resistant to browning in their regular banana varieties. This will further promote the commercialization process of GM bananas worldwide. To protect consumers’ right to know and choose, it is essential to label GM products. The first step in labeling bananas involves the detection of GM components. This requires the identification of a highly species-specific and low-copy endogenous reference gene [8]. An endogenous reference gene refers to a conserved DNA sequence with species specificity and a steady copy number that does not exhibit allelic variation [9]. With an endogenous reference gene, we can determine the presence of GM components, the percentage of GM components in a test sample, and the copy number of the sequence in the genome [10,11]. The percentage of GM is calculated as a ratio of the specific GM target with respect to an endogenous reference gene [10]. Therefore, endogenous reference genes are indispensable in GM crop research and detection [12]. To date, there have been no reports on qualitative and quantitative PCR detection methods for endogenous reference genes in banana.

To obtain a suitable endogenous reference gene for banana, this study focused on *Sucrose phosphate synthase 1* (*MaSPS1*, GenBank NO. HG996469). We sequenced the *MaSPS1* gene in different banana genotypes to compare its stability in different genomic types and determined its copy number through Southern blot analysis. The species specificity of *MaSPS1* was tested using commercialized GM crops and fruit plants. Primers and probes of *MaSPS1* were designed and tested for the establishment of a qualitative and quantitative PCR detection method. The limit of detection (LOD) of the qualitative method reached 1.14 × 10^−2^ ng, while the quantitative PCR had a LOD of 5.70 × 10^−3^ ng, with a limit of quantification (LOQ) of 1.14 × 10^−2^ ng. In summary, we identified *MaSPS1* as an endogenous reference gene for banana and established qualitative and quantitative PCR detection methods for the detection of GM banana samples.

## 2. Materials and Methods

### 2.1. Experimental Materials

The different genome types of banana materials, *Malaccensis* (genotype AA), Brazilian banana (genotype AAA), FHIA-04 (genotype AAAA), French Sombre (genotype AAB), FHIA-21 (genotype AAAB), *Musa* ABB Pisang Awak (genotype ABB), *Musa acuminata* (genotype AA), Guangxi hongjiao (genotype AAA), and CB5 (genotype AAB), were provided by Dr. Ou Sheng (Institute of Fruit Tree Research, Guangdong Academy of Agricultural Sciences). Rapeseed (*Brassica napus*, genome AACC), rice (*Oryza sativa*), papaya (*Carica Papaya*), maize (*Zea mays*), soybean (*Glycine max*), cotton (*Gossypium hirsutum*), wheat (*Triticum aestivum*), mango (*Mangifera indica*), pomelo (*Citrus maxima*), green jujube (*Ziziphus mauritiana*), lemon (*Citrus limon*), orange (*Citrus reticulata Blanco*), jackfruit (*Artocarpus heterophyllus*), watermelon (*Citrullus lanatus*), and others were supplied by our laboratory.

### 2.2. DNA Extraction and Purification

A DNA secure Plant Kit (TIANGEN BIOTECH (BEIJING) Co., Ltd., Beijing, China) was used for total DNA extraction from the plant materials. The DNA concentration and purity were evaluated using a spectrophotometer (Eppendorf, Germany) combined with agarose gel electrophoresis.

### 2.3. Primers and Probes

The candidate banana endogenous reference gene, *MaSPS1*, was obtained through database analysis and a literature review. Primers and TaqMan probes were designed using Primer Express software version 2.0 (Applied Biosystems, Foster City, CA, USA) for *MaSPS1*-specific sequences (Table 1), which were synthesized by Sangon Biotech (Shanghai) Co. Ltd., Shanghai, China.

### 2.4. MaSPS1 Sequence Analysis

Five different genotypes of banana varieties, *M. acuminata* (AA), Guangxi hongjiao (AAA), CB5 (AAB), *Musa* ABB Pisang Awak (ABB), and FHIA-21 (AAAB), were selected for amplification and sequencing with the primers SPS-1F and SPS-1R, and the sequences of the amplified *MaSPS1* gene of the five banana varieties were comparatively analyzed using SnapGene software.

### 2.5. MaSPS1 Genome Copy Number Analysis

A total of nine banana materials, as introduced in Section 2.1, were selected for Southern blot analysis. Based on the DNA sequence analysis, the restriction endonucleases *Bam*H I and *Eco*R I (Takara Bio Inc., Kusatsu-Shiga, Japan), which are absent in this sequence, were selected to digest 3 µg of total genome DNA. The DIG High Prime DNA Labeling and Detection Starter Kit II for probe labeling (item number: 11585614910, Roche, Sigma-Aldrich (Shanghai, China)) was selected. The detailed roots were measured following the previously described method [13].

### 2.6. PCR Amplification Methods

Conventional PCR amplification was performed on a PCR instrument (T100^TM^ Thermal Cycler, Bio-Rad, Hercules, CA, USA) in a final volume of 25 µL prepared as follows: 50 ng of the DNA template, 1 U of *Taq*^TM^ HS DNA polymerase (Takara Bio Inc.), 0.5 µmol/L of each of the forward and reverse primers, and 0.2 mmol/L of the dNTP Mixture (Takara Bio Inc.). The amplification steps were as follows: pre-denaturation at 95 °C for 2 min, 35 cycles of denaturation at 95 °C for 30 s, annealing at 56 °C for 30 s, extension at 72 °C for 30 s, and a final extension at 72 °C for 10 min.

Real-time fluorescence quantitative PCR (qPCR) amplification was performed on a PCR instrument (CFX Connect^TM^ Real-Time PCR Detection System, Bio-Rad, USA) in a final volume of 20 µL prepared as follows: 2× *TaKaRa Premix Ex Taq*^TM^ (Probe qPCR), 50 ng of the DNA template, 0.4 µmol/L of each of the forward and reverse primers, and 0.2 µmol/L of the probe. The amplification program was as follows: pre-denaturation at 95 °C for 3 min, 40 cycles of denaturation at 95 °C for 15 s, and annealing at 59 °C for 60 s (with the collection of fluorescence signals after annealing at 59 °C).

### 2.7. Specificity Detection of MaSPS1

The PCR templates for *MaSPS1* specificity detection included banana, *Musa* ABB Pisang Awak, and seven common crops such as rapeseed, rice, papaya, maize, soybean, cotton, and wheat, as well as thirteen common fruits such as mango, pomelo, green jujube, lemon, orange, jackfruit, watermelon, wax apple, pear, grape, peach, cantaloupe, and strawberry. The samples for the specificity tests included *Malaccensis*, Brazilian banana, FHIA-04, French Sombre, FHIA-21, *Musa* ABB Pisang Awak, *M. acuminata*, Guangxi hongjiao, and CB5.

### 2.8. Limit of Detection Test for MaSPS1

In the limit of detection test for *MaSPS1* as an endogenous reference gene, 5.70, 5.70 × 10^−1^, 1.14 × 10^−1^, 5.70 × 10^−2^, 2.85 × 10^−2^, 1.14 × 10^−2^, 5.70 × 10^−3^, 2.85 × 10^−3^, and 1.43 × 10^−3^ ng of banana genomic DNA were added to each PCR reaction, respectively.

### 2.9. Standard Curve Construction and Limit of Quantification Test for qPCR

In the standard curve construction of qPCR, the banana genomic DNA of 1.14 × 10^2^, 1.14 × 10^1^, 1.14 × 10^0^, 1.14 × 10^−1^, 1.14 × 10^−2^, and 5.70 × 10^−3^ ng was added to each PCR reaction. The standard curve equation is y = ax + b, where the Ct value is used as the *y*-axis and the logarithm of the copy number is used as the *x*-axis. The slope of the standard curve must be in the range of −3.6 to −3.1, the R^2^ value should be greater than or equal to 0.98, and the amplification efficiency must fall between 90% to 110%.

## 3. Results

### 3.1. Sequence Analysis of MaSPS1

**Figure 1:**
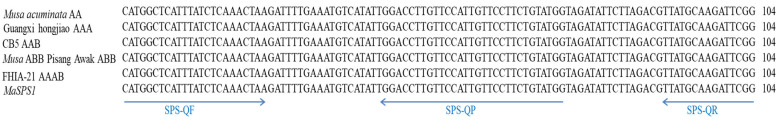
Sequence analysis of the *MaSPS1* gene. Note: The position of the forward (SPS-QF) and reverse (SPS-QR) primers and fluorescence probes (SPS-QP) of the amplified sequence are indicated separately in the sequence. Five different banana varieties with distinct genotypes, *M. acuminata* (AA), Guangxi hongjiao (AAA), CB5 (AAB), *Musa* ABB Pisang Awak (ABB), and FHIA-21 (AAAB), were selected for analysis.

To screen and identify the endogenous reference gene in banana, we selected *MaSPS1* as the candidate gene. And we chose five different banana varieties with distinct genotypes, including *M. acuminata* (AA), Guangxi hongjiao (AAA), CB5 (AAB), *Musa* ABB Pisang Awak (ABB), and FHIA-21 (AAAB), to sequence and analyze the conserved region. The results indicated that the amplified sequence was highly conserved. There were no differences observed between *MaSPS1* and these five different banana varieties in the 104 bp of the PCR amplification sequence region (Figure 1). This suggests that this region sequence of *MaSPS1* is highly conserved in the different genotypes of banana genomes and can be used as the amplification region in the method construction process.

### 3.2. Specificity Test for MaSPS1

**Figure 2:**
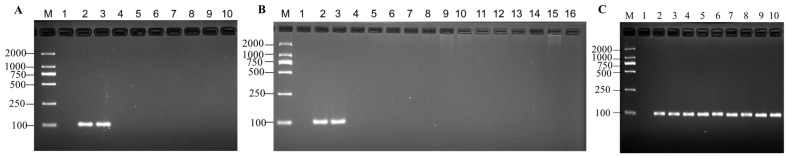
Conventional PCR to verify *MaSPS1* gene specificity. (**A**) Specific amplification of *MaSPS1* in crops. 1~10: blank control, banana, *Musa* ABB Pisang Awak, rapeseed, rice, papaya, maize, soybean, cotton, and wheat, respectively. The 104 bp target band was only amplified in the banana and *Musa* ABB Pisang Awak reactions. (**B**) Specific amplification of *MaSPS1* in fruits. 1~16: blank control, banana, *Musa* ABB Pisang Awak, mango, grapefruit, green jujube, lemon, orange, jackfruit, watermelon, wax apple, pear, grape, peach, cantaloupe, and strawberry, respectively. The 104 bp target band was only amplified in the banana and *Musa* ABB Pisang Awak reactions. (**C**) Specific amplification of *MaSPS1* in the different genotypes of banana. 1~10: blank control, *Malaccensis* (AAA), Brazilian banana (AAA), FHIA-04 (AAAA), French Sombre (AAB), FHIA-21 (AAAB), *Musa* ABB Pisang Awak (ABB), *M. acuminata* (AAA), Guangxi hongjiao (AAA), and CB5 (AAB), respectively. All of these banana materials were able to amplify a specific target band of 104 bp.

**Figure 3:**
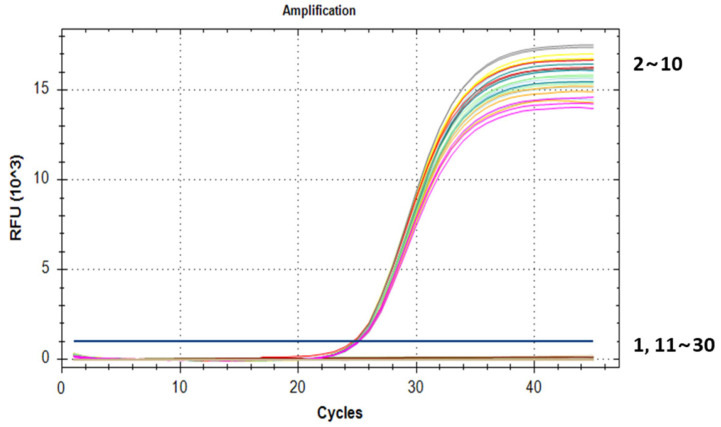
Real-time fluorescence quantitative PCR (qPCR) to verify *MaSPS1* specificity. 1: blank control; 2~10: Malaccensis, Brazilian banana, FHIA-04, French Sombre, FHIA-21, *Musa* ABB Pisang Awak, *M. acuminata*, Guangxi hongjiao, and CB5 had amplification signals; 11~30: rapeseed, rice, papaya, maize, soybean, cotton, wheat, mango, grapefruit, green jujube, lemon, orange, jackfruit, watermelon, wax apple, pear, grape, peach, cantaloupe, and strawberry.

To identify the specificity of MaSPS1 in the banana genome, we selected commercially available GM crops such as rapeseed, maize, soybean, cotton, and papaya, as well as major crops, including wheat and rice, to amplify MaSPS1. The electrophoresis results showed that the 104 bp target band was amplified in banana and Musa ABB Pisang Awak, but not in the other crops (Figure 2A). Moreover, the fluorescence signal only appeared in the banana and Musa ABB Pisang Awak reactions during qPCR testing (Figure 3; Table 2). Bananas are both a tropical and subtropical form of fruit. Therefore, we also tested whether MaSPS1 could be amplified in mango, pomelo, green jujube, lemon, orange, jackfruit, watermelon, wax apple, pear, grape, peach, cantaloupe, and strawberry. Similarly, the 104 bp target band for MaSPS1 was only amplified in the banana and Musa ABB Pisang Awak but not in the other fruits (Figure 2B). The results of qPCR testing also showed that MaSPS1 had amplification signals only in bananas, and no effective amplification signals were detected in other fruits, which was consistent with the results of conventional PCR electrophoresis (Figure 3, Table 2).

To test the stability and specificity of MaSPS1 in the different banana genotypes, we conducted PCR testing and gel electrophoresis on nine banana materials, including the AA, AAA, AAAA, AAB, AAAB, and ABB genotypes. The results revealed that all of these banana materials were able to amplify a target band of 104 bp (Figure 2C). The results of the qPCR also revealed that the Ct values of MaSPS1 were similar in the different genotypes of the bananas tested, from 24.79 to 25.15 (Figure 3, Table 2). This indicated that the gene fragment is specific to the banana materials tested.

### 3.3. Determination of the Copy Number of MaSPS1 Using Southern Blot Analysis

**Figure 4:**
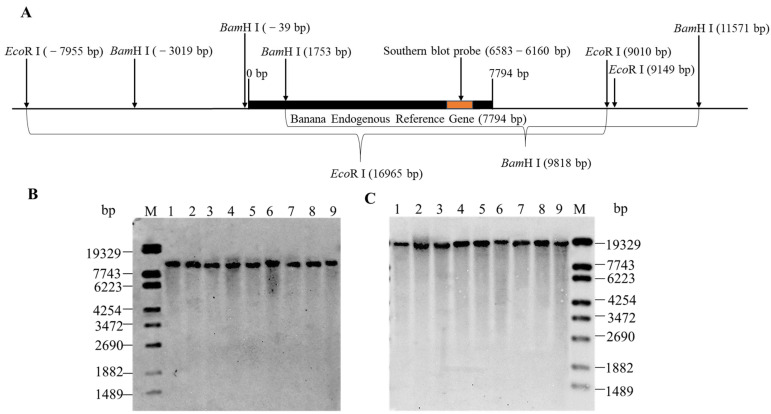
Southern blot analysis the copy number of *MaSPS1* in the banana genome. (**A**) Enzymatic cleavage site analysis of the *MaSPS1* genomic sequence. Southern blot with *Bam*H I (**B**) and *Eco*R I (**C**) digestion of banana genomic DNA. The hybridization band was about 10 kb when *Bam*H I digestion was employed for all nine banana varieties in (**B**). The hybridization band was about 17 kb when *Eco*R I digestion was utilized (**C**). 1~9: *Malaccensis* (AA), Brazilian banana (AAA), FHIA-04 (AAAA), French Sombre (AAB), FHIA-21 (AAAB), *Musa* ABB Pisang Awak (ABB), *M. acuminata* (AAA), Guangxi hongjiao (AAA), and CB5 (AAB).

To ascertain the copy number of the *MaSPS1* gene in the banana genome, we analyzed the genomic sequence of *MaSPS1* and found that there are no *Bam*H I and *Eco*R I recognition sites (Figure 4A). Subsequently, we performed a Southern blot analysis after digesting the banana genomic DNA with *Bam*H I and *Eco*R I. The results revealed that all nine banana varieties exhibited a distinct approximately 10 kb hybridization band when *Bam*H I digestion was employed (Figure 4B). Likewise, all nine banana varieties displayed prominent hybridization bands at about 17 kb when *Eco*R I digestion was utilized (Figure 4C). These findings corroborate the results of the sequencing analysis (Figure 4A), indicating that the *MaSPS1* gene exists as a single copy within the banana genome.

### 3.4. Limit of Detection Test

**Figure 5:**
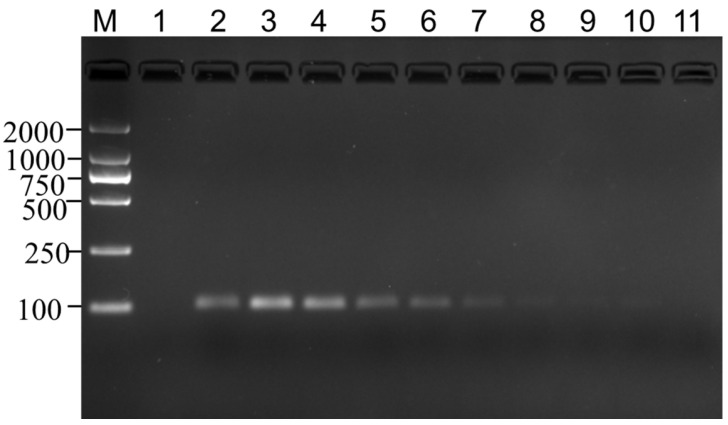
Conventional PCR LOD test for *MaSPS1*. 1~11: blank control, positive control, 5.70 × 10^0^, 5.70 × 10^−1^, 1.14 × 10^−1^, 5.70 × 10^−2^, 2.85 × 10^−2^, 1.14 × 10^−2^, 5.70 × 10^−3^, 2.85 × 10^−3^, and 1.43 × 10^−3^ ng of banana genomic DNA. A clear 104 bp target band was seen when the template was 1.14 × 10^−2^ ng and above.

To validate the LOD of the banana endogenous reference gene detection method, we first carried out conventional PCR detection. The results revealed that when the genomic DNA template was 1.14 × 10^−2^ ng, a clear target band could be seen in the electrophoresis test (equivalent to about 20 copies). Moreover, the brightness of the target band increased with the increase in the DNA template (Figure 5), indicating that the LOD of the conventional PCR detection method for the MaSPS1 endogenous reference gene is around 20 copies.

**Figure 6:**
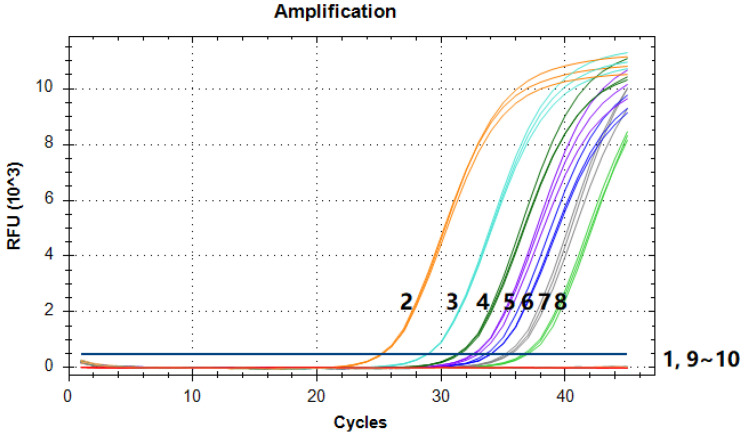
qPCR LOD test for *MaSPS1*. 1~10: blank control, 5.70 × 10^0^, 5.70 × 10^−1^, 1.14 × 10^−1^, 5.70 × 10^−2^, 2.85 × 10^−2^, 1.14 × 10^−2^, 5.70 × 10^−3^, 2.85 × 10^−3^, and 1.43 × 10^−3^ ng of banana genomic DNA. Amplification curves could be obtained when the template was 5.70 × 10^−3^ ng and above.

To further validate the LOD of the qPCR method for *MaSPS1* as an endogenous reference gene, we performed fluorescence PCR with gradient concentrations of DNA. The results showed that when the DNA template quantity was 5.70 × 10^−3^ ng (about 10 copies) and above, typical amplification curves could be obtained. This suggested that the LOD of the quantitative PCR method for the *MaSPS1* endogenous reference is around 10 copies (Figure 6; Table 3). Furthermore, the amplification curves between the same concentrations overlapped significantly and had high repeatability, indicating that the method has high stability and reliability.

### 3.5. Construction of a Standard Curve and the LOQ Test

**Figure 7:**
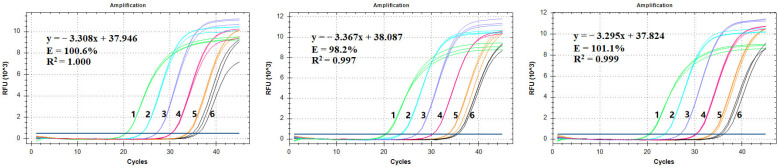
qPCR repeatability and stability test for *MaSPS1*. (**A**–**C**) are the amplification curves and standard curves of the qPCR test under different models of three operators; 1~6: 1.14 × 10^2^, 1.14 × 10^1^, 1.14 × 10^0^, 1.14 × 10^−1^, 1.14 × 10^−2^, and 5.70 × 10^−3^ ng of banana genomic DNA.

To validate the established qPCR detection method, we first diluted template genomic DNA with gradient concentrations. The amplification results for the five gradients of template DNA showed that the three technical repetitions of each sample were highly consistent (Figure 7A, Table 4). According to the standard curve, the amplification efficiency of MaSPS1 was 100.6% and the linear correlation coefficient R^2^ was 1.000 (Figure 7A), indicating that the established qPCR method could meet the quantitative detection requirements. Based on the results of gradient DNA detection, the relative deviation was 10.37% when the genomic template quantity was 1.14 × 10^−2^ ng (Table 4). Therefore, the LOQ for this method is about 1.14 × 10^−2^ ng (equivalent to about 20 copies).

### 3.6. Repeatability and Stability Tests for the qPCR Method

To assess the stability and reliability of the qPCR detection method established in this study, we conducted independent tests with different operators and different instruments. The results demonstrated that the amplification curves of the five gradient concentration standard samples were very similar (Figure 7). The amplification efficiencies were 100.6%, 98.2%, and 101.1%, and the linear correlation coefficients R^2^ were all higher than 0.99 (Figure 7; Table 5). Similar results were obtained for samples of the same concentration, with copy number quantification deviation biases all below 10% (Table 6). For the LOD of 5.70 × 10^−3^ ng banana genomic DNA, there was a relatively larger deviation in the amplification curve, but signals were still consistently detected (Figure 7; Table 6). These results indicated that the established qPCR method shows good repeatability and stability.

## 4. Discussion

Since the commercialization of GM plants, they have achieved rapid development over a span of more than 20 years, with significant growth in cultivation area and production value, leading to remarkable economic and ecological benefits [14]. Currently, a large area of GM crops is cultivated globally including maize, soybean, rapeseed, and cotton. GM papaya and alfalfa are also widely used in some countries [15]. Due to the substantial advantages of GM technology, it has also been successfully applied in vegetable and fruit research [16,17]. Bananas, being important fruits and food crops, have faced significant challenges due to the devastating impact of wilt disease, which has limited their yield and quality. GM technology, including gene-editing techniques, represents the most efficient method to combat Fusarium wilt effectively. In April 2023, gene-edited bananas in the Philippines received regulatory exemptions and they were set to be grown and sold alongside conventional bananas. This marks the future availability of various GM banana varieties. Therefore, the identification and study of endogenous reference genes, which are essential for GM crop research and detection, have become urgent and necessary.

Endogenous reference genes are necessary for the detection of GM plants. The amplification of endogenous reference genes is essential for confirming the identity of GM crops and calculating the content of GM components [10,11]. Currently, several endogenous reference genes have been developed and reported for various crops, including HMG I/Y in rapeseed [18], zSSIIb in maize [19], papain in papaya [20], Lectin in soybean [21], and GhPP4A14 in cotton [22]. These genes have been applied in multiple detection systems and have played pivotal roles in GM detection work. For example, the Lectin gene is used as a detection standard in both China and the European Union [23,24]. In this study, we identified MaSPS1, a highly conserved Sucrose phosphate synthase gene in banana. It demonstrated high conservation among different banana genotypes, and Southern blot analysis confirmed that it exists as a single copy in all detected banana genomes and is identical among different genotypes. Therefore, MaSPS1 possesses typical characteristics of an endogenous reference gene.

In GM component detection, the LOD is a crucial parameter that must be determined. According to literature reports, the LOD for conventional PCR is generally around 20 copies, while for qPCR, it is around 10 copies [25,26]. The LOD of the detection system established in this study is consistent with the reported results. The constructed standard curve for qPCR complies with the quantitative requirements, with a LOQ of 20 copies. Furthermore, this study tested the stability of the detection system with different operators and different machines. The results indicated that the detection limit was consistent across different operators and machine models. Overall, this study demonstrates that MaSPS1 is a stable and reliable endogenous reference gene suitable for qualitative and quantitative PCR detection in GM bananas.

## Figures and Tables

**Table 1:** Sequence information of the primers and probes used in the study.

Purpose	Primers/Probe Name	Sequence (5′–3′)	Amplicon (bp)
Sequencing analysis	SPS-1F	CAAACAACGGGAAGCCTTTTTC	1170
	SPS-1R	CCTTTTCGCCTTCTGATAGTTC	
Southern blot probe	SPS-2F	CTGATAATTTGTTCGTAGACC	423
	SPS-2R	CCGAATCTTGCATAACGTCTAA	
PCR amplification	SPS-QF	CATGGCTCATTTATCTCAAACTAA	104
	SPS-QR	CCGAATCTTGCATAACGTCTAA	
	SPS-QP	FAM-GGACCTTGTTCCATTGTTCCTTCTGTATGG-BHQ1	

**Table 2:** Ct values of *MaSPS1* from the plant species in qPCR.

Plant Species	Ct Value			Mean Ct	SD	RSD
	Repeat 1	Repeat 2	Repeat 3			
Blank control	N/A	N/A	N/A			
*Malaccensis*	25.03	24.77	24.58	24.79	0.23	0.91%
Brazilian banana	25.20	25.10	25.15	25.15	0.05	0.20%
FHIA-04	25.05	25.11	24.96	25.04	0.08	0.30%
French Sombre	25.12	25.03	25.04	25.06	0.05	0.20%
FHIA-21	25.05	24.97	24.94	24.99	0.06	0.23%
*Musa* ABB Pisang Awak	25.16	25.00	25.07	25.08	0.08	0.32%
*M. acuminata*	24.70	25.08	24.79	24.86	0.20	0.80%
CB5	24.99	24.98	25.11	25.03	0.07	0.29%
Guangxi hongjiao	24.92	24.76	24.81	24.83	0.08	0.33%
Rapeseed	N/A	N/A	N/A			
Rice	N/A	N/A	N/A			
Papaya	N/A	N/A	N/A			
Maize	N/A	N/A	N/A			
Soybean	N/A	N/A	N/A			
Cotton	N/A	N/A	N/A			
Wheat	N/A	N/A	N/A			
Mango	N/A	N/A	N/A			
Grapefruit	N/A	N/A	N/A			
Green jujube	N/A	N/A	N/A			
Lemon	N/A	N/A	N/A			
Orange	N/A	N/A	N/A			
Jackfruit	N/A	N/A	N/A			
Watermelon	N/A	N/A	N/A			
Wax apple	N/A	N/A	N/A			
Pear	N/A	N/A	N/A			
Grape	N/A	N/A	N/A			
Peach	N/A	N/A	N/A			
Cantaloupe	N/A	N/A	N/A			
Strawberry	N/A	N/A	N/A			

**Table 3:** Copy number test for *MaSPS1* in qPCR.

DNA Concentration		Signal Rate (Positive Signals)	Copy Number		
ng/reaction	Copies/reaction		Mean	SD	RSD
5.70 × 10^0^	10,000	3/3	9351.2	353.12	3.78%
5.70 × 10^−1^	1000	3/3	987.3	14.93	1.51%
1.14 × 10^−1^	200	3/3	231.5	14.71	6.36%
5.70 × 10^−2^	100	3/3	101.9	14.88	14.59%
2.85 × 10^−2^	50	3/3	51.7	8.29	16.03%
1.14 × 10^−2^	20	3/3	20.8	2.48	11.94%
5.70 × 10^−3^	10	3/3	8.8	1.06	12.07%
2.85 × 10^−3^	5	0/3	N/A		
1.43 × 10^−3^	2.5	0/3	N/A		

**Table 4:** LOQ test for *MaSPS1* in qPCR.

DNA Concentration		Ct Value						Copy Number		
ng/reaction	Copies/ reaction	Repeat 1	Repeat 2	Repeat 3	Mean	SD	RSD	Mean	SD	RSD
1.14 × 10^2^	200,000	20.39	20.34	20.40	20.38	0.03	0.16%	204,983.7	4613.07	2.25%
1.14 × 10^1^	20,000	23.74	23.76	23.82	23.77	0.04	0.18%	19,269.5	554.91	2.88%
1.14 × 10^0^	2000	27.09	27.06	27.02	27.06	0.04	0.13%	1959.8	47.99	2.45%
1.14 × 10^−1^	200	30.30	30.27	30.20	30.26	0.05	0.17%	211.3	7.60	3.59%
1.14 × 10^−2^	20	33.60	33.57	33.85	33.67	0.15	0.46%	19.7	2.04	10.37%
5.70 × 10^−3^	10	36.04	34.73	35.39	35.39	0.66	1.85%	6.4	2.83	44.52%

**Table 5:** qPCR standard curve repeatability test for *MaSPS1.*

Test Number	Amplification Efficiency	R^2^	Slope	Y-Intercept
Test 1	100.6%	1.000	−3.308	37.946
Test 2	98.2%	0.997	−3.367	38.087
Test 3	101.1%	0.999	−3.295	37.824

**Table 6:** qPCR copy number repeatability test for *MaSPS1.*

DNA Concentration		Test 1 Copy Number		Test 2 Copy Number		Test 3 Copy Number	
ng/reaction	Copies/ reaction	Mean	Bias	Mean	Bias	Mean	Bias
1.14 × 10^2^	200,000	204,755.5	2.4%	185,916.0	7.0%	193,029.0	3.5%
1.14 × 10^1^	20,000	19,252.3	3.7%	21,091.1	5.5%	21,280.5	6.4%
1.14 × 10^0^	2000	1958.4	2.1%	2107.2	5.4%	1996.4	0.2%
1.14 × 10^−1^	200	211.2	5.6%	210.7	5.4%	195.5	2.2%
1.14 × 10^−2^	20	19.6	1.8%	19.6	2.2%	20.3	1.4%
5.70 × 10^−3^	10	6.4	36.4%	6.7	33.0%	4.8	51.9%

## Data Availability

The data presented in this study are available in [The Identification of the Banana Endogenous Reference Gene *MaSPS1* and the Construction of Qualitative and Quantitative PCR Detection Methods].
